# Supplementary material for: Engineered Small Extracellular Vesicles as a FGL1/PD‐L1 Dual‐Targeting Delivery System for Alleviating Immune Rejection
Source: Adv Sci (Weinh). 2021 Nov 5;9(3):2102634. doi: 10.1002/advs.202102634 (PMC8787398; doi:10.1002/advs.202102634)
Supplement: Supplementary file 1 — Supporting Information [file ADVS-9-2102634-s001.pdf]

© 2021 Wiley-VCH GmbH

ADVANCED SCIENCE
Open Access

Supporting Information

for *Adv. Sci.,* DOI: 10.1002/advs.202102634

Engineered Small Extracellular Vesicles as a FGL1/PD-L1 Dual-targeting Delivery System for Alleviating Immune Rejection

Hsiang-i Tsai, Yingyi Wu, Xiaoyan Liu, Zhanxue Xu, Longshan Liu, Changxi Wang, Huanxi Zhang, Yisheng Huang, Linglu Wang, Weixian Zhang, Dandan Su, Fahim Ullah Khan, Xiaofeng Zhu, Rongya Yang, Yuxin Pang, John E. Eriksson, Haitao Zhu, Dongqing Wang, Bo Jia, Fang Cheng*, and Hongbo Chen**

Supporting Information

Engineered small extracellular vesicles as a FGL1/PD-L1 dual-targeting delivery system for alleviating immune rejection

Hsiang-i Tsai, Yingyi Wu, Xiaoyan Liu, Zhanxue Xu, Longshan Liu, Changxi Wang, Huanxi Zhang, Yisheng Huang, Linglu Wang, Weixian Zhang, Dandan Su, Fahim Ullah Khan, Xiaofeng Zhu, Rongya Yang, Yuxin Pang, John E. Eriksson, Haitao Zhu, Dongqing Wang, Bo Jia, Fang Cheng*, and Hongbo Chen**

Dr. H.I. Tsai[+], Y. Y. Wu, X. Y. Liu, Z. X. Xu, L. L. Wang, W. X. Zhang, D. D. Su, Prof. F. Cheng, Prof. H. B. Chen
School of Pharmaceutical Sciences (Shenzhen)
Sun Yat-sen University
Shenzhen 518107, P.R. China
E-mail: chengf9@mail.sysu.edu.cn, chenhb7@mail.sysu.edu.cn

Prof. H.T. Zhu, Prof. D.Q. Wang
Department of Medical Imaging
The Affiliated Hospital of Jiangsu University
Zhenjiang 212001, China

Dr. H. X. Zhang, Prof. L. S. Liu, Prof. C. X. Wang
Organ Transplant Center
The First Affiliated Hospital, Sun Yat-sen University
58 Zhongshan 2nd Road, Guangzhou, Guangdong 510080, China

Dr. Y. S. Huang, Dr. B. Jia
Department of Oral Surgery
Stomatological Hospital, Southern Medical University
Guangzhou 510280, Guangdong, PR China
E-mail: dentist-jia@163.com

Dr. F. U. Khan
Zoology Department UST
Bannu Kp 28100, Pakistan

Dr. X. F. Zhu, Prof. Y.X Pang
School of Traditional Medicine Materials Resource, Guangdong Pharmaceutical University
Yunfu 527322, Guangdong, China.

Prof. R.Y. Yang
Department of Dermatology, The Seventh Medical Center of PLA General Hospital, Peking 100010, China

Prof. J. Eriksson
Cell Biology, Biosciences, Faculty of Science and Engineering
Åbo Akademi University,
FI-20520, Turku, Finland

[+] Present address: Department of Medical Imaging
The Affiliated Hospital of Jiangsu University
Zhenjiang 212001, China

H. Tsai, Y. Y. Wu and X. Y. Liu contributed equally to this work.
B. Jia, F. Cheng, and H. B. Chen are corresponding authors

Supporting Information

Supplement Figures

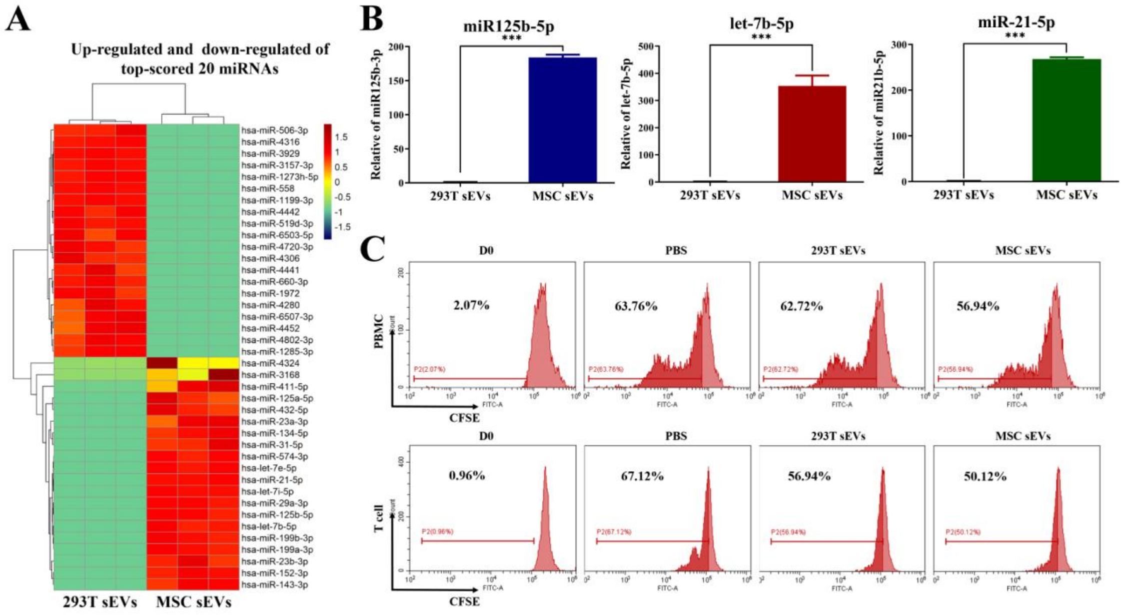

Figure S1. MSC sEVs has a negative immune regulation function. (related to Figure 2)

A) Heat map of miRNAs targeting these immune genes created using 4,872 Immunologic Signature Gene Sets provided by the GSEA website, $n = 3$**.** B) Real time RT-PCR confirmation of miR125b-5p, let-7b-5p and miR-21-5p up-regulated by miRNA-sequencing, $n = 3$. Error bar, mean ± SEM. P-values are calculated using student T-test. $*P < 0.05$, $**P < 0.01$, $***P < 0.001$**.** C) Inhibition of PBMC and $CD3^{+}$ T cells over a 5 day period by different sEVs groups using CFSE staining. PBMCs or T cells were stimulated with plate-bound CD3 (10 μg/mL) and IL-2 (2 ng/mL), then treated with the indicated different types of sEVs (50 μg/mL). Cell proliferation was detected by CFSE at 5 days post-treatment, respectively. NC group: treated with PBS. D0 group: CFSE assay at 0 days post-treatment. CFSE staining was analyzed by flow cytometry.

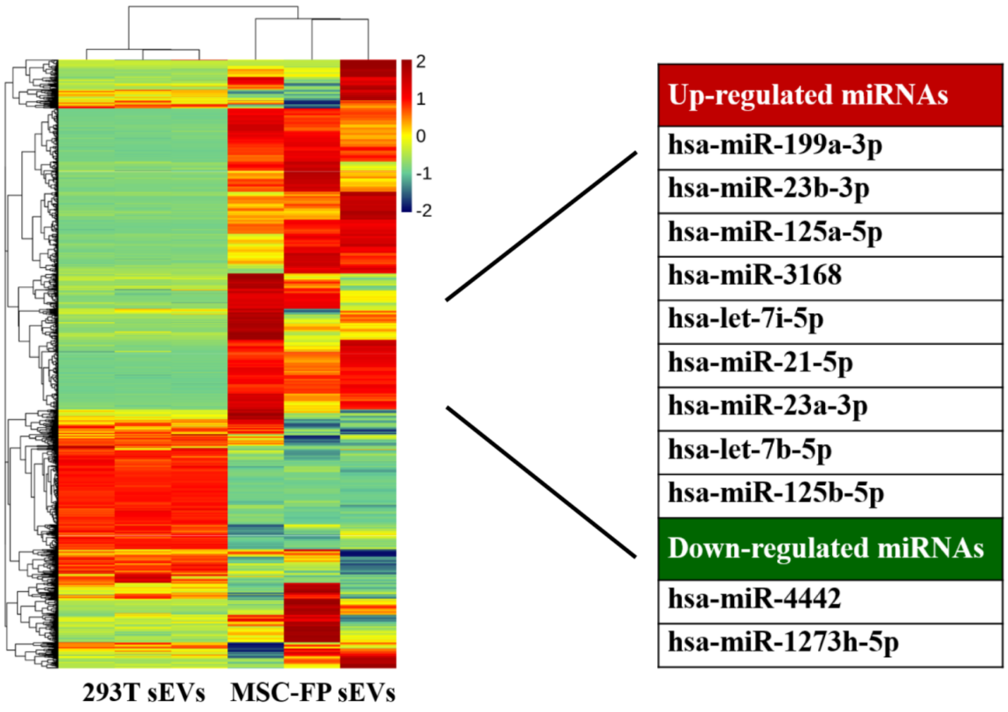

Figure S2. Modification couldn't affect the negative immune regulation function of the miRNAs in exosomes. (related to Figure 2)

Heat map of miRNAs in HEK-293T sEVs and MSC-FP sEVs targeting whole genome provided by the GSEA website, n = 3**.**

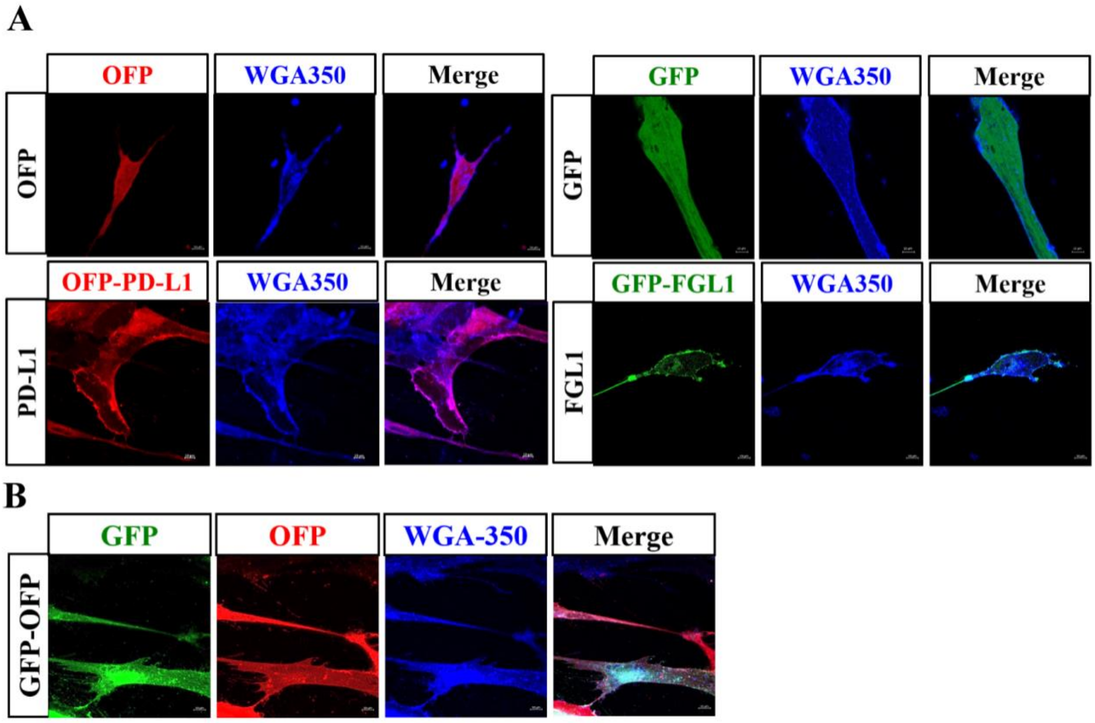

Figure S3. Construction of MSC cell line stably expressing GFP or OFP and GFP/OFP. (related to Figure 3)

A) Confocal images indicated the expression of GFP or OFP in MSC cells. B) Confocal images indicated the expression of GFP and OFP in MSC cells. WGA-Alexa-350 was used to stain cell membranes. Scale bar: 10 μm.

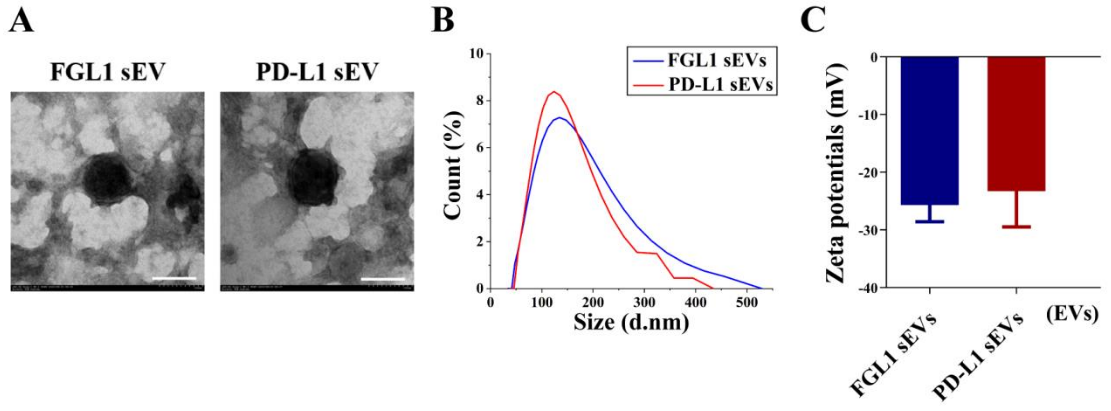

Figure S4. Establishment and characterization of FGL1 sEVs and PD-L1 sEVs. (related to Figure 3)

A-C) The TEM images (A), size distribution (B), and the Zeta potential (C) of purified sEVs from MSC-FGL1 and MSC-PD-L1 cells, *n* = 3. (scale bar: 100 nm).

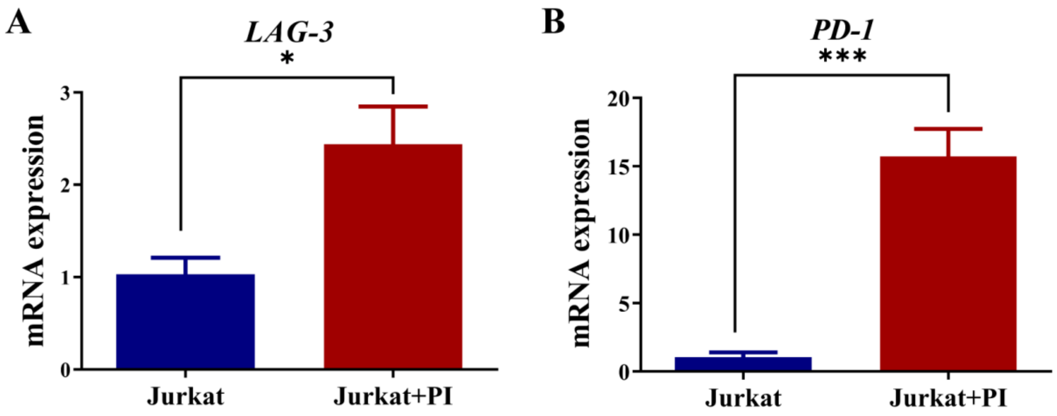

Figure S5. Upregulated PD-1 and LAG-3 mRNA levels of PBMC and Jurkat cell after PI stimulation. (related to Figure 4)

A-B) Expression of LAG-3 (A) and PD-1 (B) mRNA levels in Jurkat cells upon P/I stimulation as detected by qPCR, $n = 3$. Error bar, mean ± SEM. P-values are calculated using student T-test. *$p < 0.05$, **$p < 0.05$, ***$p < 0.001$.

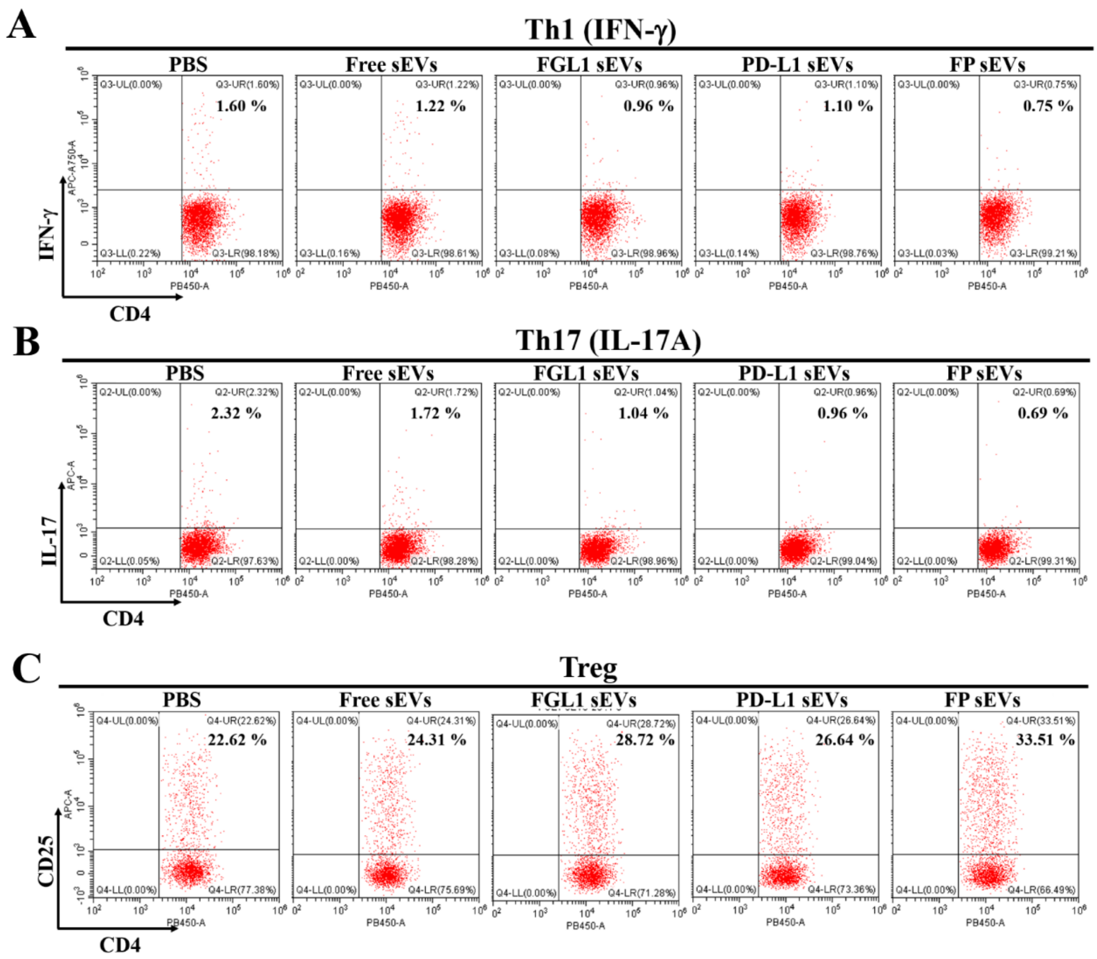

Figure S6. FGL1/PD-L1 sEVs could affect Th1, Th17 and Treg differentiation. (related to Figure 4)

A-C) Flow cytometry analysis of $CD4^+$ IFN-γ^+ Th1 (A), $CD4^+$ IL-$17A^+$ Th17 (B) and $CD4^+$ $CD25^+$ Treg (C) expression in P/I-stimulated $CD3^+$ T cells treated with or without different groups of sEVs.

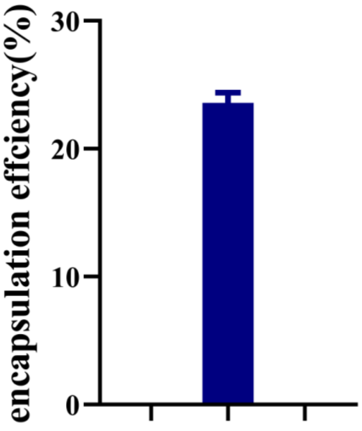

Figure S7. Encapsulation rate of FGL1/PD-L1 sEVs loaded with FK506. (related to Figure 4)

FK506 was encapsulated in the FGL1/PD-L1 sEVs by electroporation, and its encapsulation rate was detected by UV-VIS at 300 nm (*n* = 3).

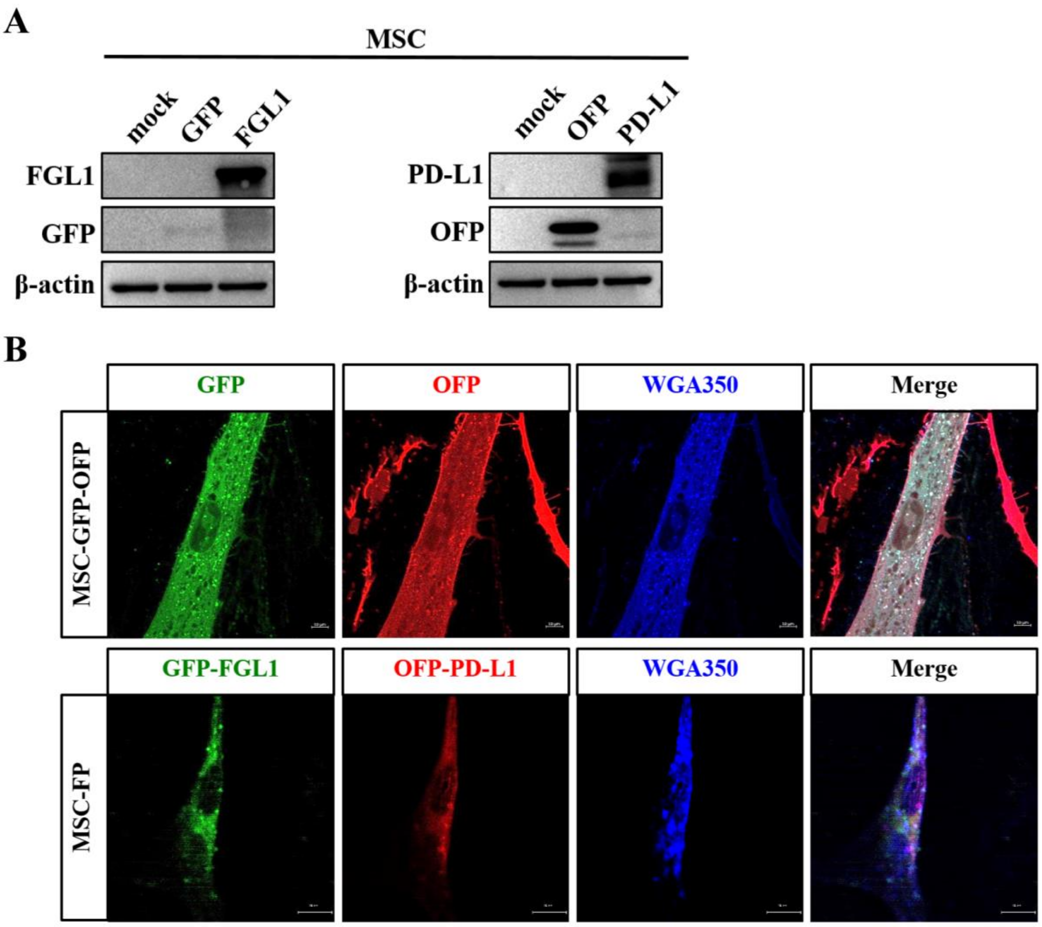

Figure S8. Establishment of MSC cells expressing mouse-FGL1/PD-L1. (related to Figure 5)

A) Western blotting for mouse-PD-L1-OFP and mouse-FGL1-GFP in the whole cell lysate in MSCs. B) Confocal images indicate the expression of mouse-PD-L1-OFP, mouse-FGL1-GFP and mouse-PD-L1-OFP/mouse-FGL1-GFP in MSCs. Cell membranes stained with WGA Alexa 350. Scale bar: 10 μm.

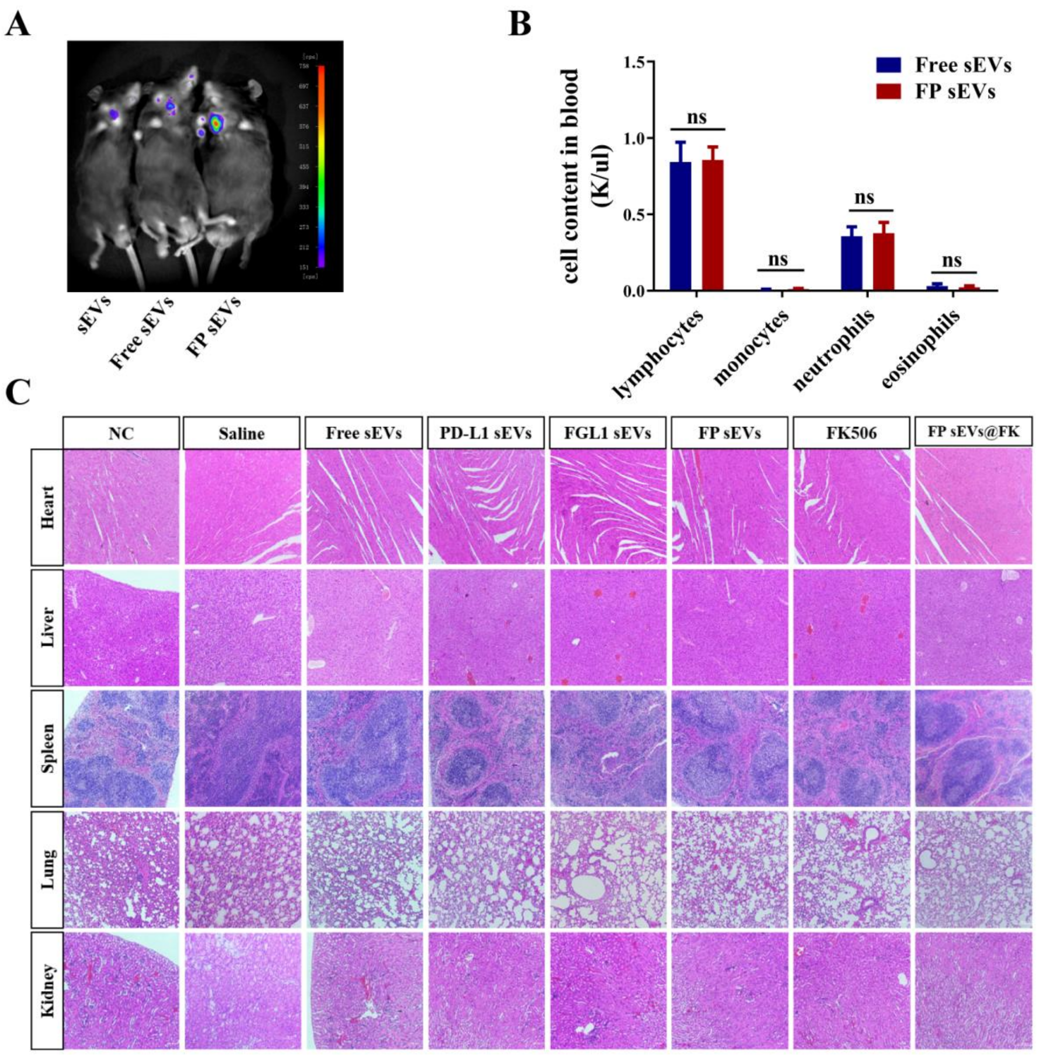

Figure S9. Bioluminescence imaging and toxicity test of FGL1/PD-L1 sEVs in mice. (related to Figure 5)

A) *In vivo* Bioluminescence imaging of sEVs, Free sEVs, and FP sEVs originating from MSC cells via tail vein injection. sEVs: injected with MSC-sEVs. Free sEVs: injected with vector sEVs. B) Complete blood count test (CBC test). Mice were injected with Free sEVs or FP sEVs via tail vein injection for 14 days for whole blood analysis, $n = 5$. Error bar, mean ± SEM. *P*-values are calculated using student T-test. ns: not significant. C) Histological images obtained from the hearts, livers, spleens and kidneys of mice treated with NC, saline, or different groups of EVs for 14 days post-injection as compared to the NC group. Scale bar: 100 μm.

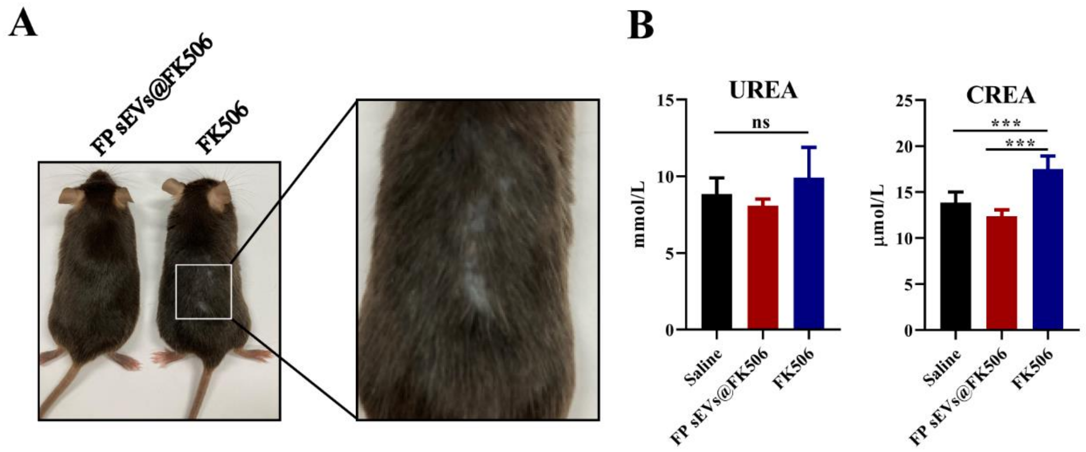

Figure S10. The appearance and biochemical analysis of FK506 and FP sEVs @ FK506 in heart allogenic mice. (related to Figure 5)

A) Images highlighting the hair loss of heart allogenic mice after injecting with FK506 or FP sEVs@FK506. B) Blood samples were collected after injecting with saline, FK506 or FP sEVs@FK506 to measure creatinine (CREA) and urea nitrogen (UREA) by automatic blood analyzer. *n* = 5. Error bar, mean ± SEM. *P < 0.05, **P < 0.01, ***P < 0.001.

Supplementary Tables

Table S1. Top-scored negative immunoregulation miRNAs between HEK-293T sEVs and MSC-FP sEVs.

**Up-regulated miRNAs**	**ID**	***P* VALUE**	**Function**
hsa-miR-199a-3p[1]	MIMAT0000232	1.45713E-08	Inhibition of inflammation, immunity and resistance to kidney damage
hsa-miR-23b-3p[2]	MIMAT0000418	2.43588E-05	Effective treatment of rheumatoid arthritis
hsa-miR-125a-5p[3]	MIMAT0000443	0.008456264	Promote the generation of M2 type macrophages
hsa-miR-3168[4]	MIMAT0015043	8.46957E-25	Suppress the immune system and relieve asthma
hsa-let-7i-5p[5]	MIMAT0000415	1.19388E-33	Inhibition of IL - 6
hsa-miR-21-5p[6]	MIMAT0000076	0.000657411	Inhibits the immune function of dendritic cells
hsa-miR-23a-3p[7]	MIMAT0000078	3.35175E-05	Upregulate the expression of PD-L1 to inhibit T cells function
hsa-let-7b-5p[8]	MIMAT0000063	4.70362E-27	Inhibit the expression of inflammatory cytokines in macrophages
hsa-miR-125b-5p[9]	MIMAT0000423	1.37316E-88	Inhibition of inflammation, immunity and resistance to kidney damage
**Down-regulated miRNAs**	**ID**	***P* VALUE**	**Function**
hsa-miR-4442[10]	MIMAT0018960	1.2899E-122	Downregulation inhibits inflammation and relieves dermatomyositis
hsa-miR-1273h-5p[11]	MIMAT0030415	1.0546E-166	Increased in the PBMCs of SLE patients

Table S2. Description of T cell related factors

**Factor**	**Function description**	**Reference**
IL-2	*Promote the differentiation of $CD4^+$T cells and activate Treg cells*	[12]
IL-18	*Promote the differentiation of $CD4^+$T cells*	[13]
IL-22	*Promote the differentiation of $CD4^+$T cells*	[14]
IL-10	*Activate Treg cells*	[15]
IL-5	Regulating the differentiation, growth and activation of eosinophils	[16]
IL-17A	*Promote the differentiation of $CD4^+$T and $CD8^+$ cells*	[14, 17]
IL-21	*Inhibit the proliferation of Treg cells*	[18]
IL-12	*Promote the differentiation of $CD4^+$T cells*	[18a, 19]
IL-6	Regulator of Treg/Th17 balance	[20]
CCL1	Suppressive function of Tregs	[21]
ID3	Control of the differentiation of regulatory T cells and TH17 cells	[22]
ID1	Promotes T Regulatory Cell Differentiation	[23]
TNFRSF12	Promotes T Regulatory Cell Differentiation	[24]
BMPR1B	Development and Activation of $CD4^+$ T Cells	[25]
CX3CL1	Chemotactic activity for monocytes, NK cells, and T cells	[26]
IL11	Promote the proliferation and differentiation of $CD4^+$T cells	[27]
ILF	Regulator of Treg/Th17 balance	[28]

CXCL13	*Activate* Tregs *cells*	[29]
CCL4	*Inhibit the proliferation of* $CD8^+$ *cells*	[30]
IL12RB2	Promotes Th1 Cell Differentiation	[31]
TNFSF14	*Activate NK T cells*	[32]
IDO1	*Activate* Tregs *cells*	[33]
IL36A	*Activate T cells*	[34]
IL1RN	*Activate* Th17 *cells*	[35]
IL12A	Promotes $CD8^+$ Cell Differentiation	[36]
IL21	Promotes $CD8^+$ Cell Differentiation	[37]
TNFRSF8	Promotes Th17 Cell Differentiation	[38]
TNFRSF12A	Promotes *the proliferation of T cells*	[39]
EDA2R	*Inhibit the* Differentiation *of Th17 cells*	[40]
TSLP	*enhances the function of helper type 2 cells*	[41]

Table S3. The demographic clinical characteristics in kidney transplant patients.

Variables	Total (n = 19)	ABMR (n = 6)	TCMR (n = 6)	Stable (n = 7)	*P*
Donors					
Types, n (%)					**0.045**
DD	11 (58)	2 (33)	6 (100)	3 (43)	
LD	8 (42)	4 (67)	0 (0)	4 (57)	
Recipients					
Gender, n (%)					**0.037**
Female	9 (47)	1 (17)	2 (33)	6 (86)	
Male	10 (53)	5 (83)	4 (67)	1 (14)	
Age (year), Mean ± SEM	36.96 ± 9.30	42.10 ± 10.44	37.43 ± 10.60	32.14 ± 4.61	0.156
Weight (kg), Mean ± SEM	52.0 ± 12.2	59.3 ± 16.1	50.2 ± 7.2	47.3 ± 10.2	0.191
Previous_transplantation, n (%)					0.48
0	16 (84)	6 (100)	4 (67)	6 (86)	
1	3 (16)	0 (0)	2 (33)	1 (14)	
Days_from_transplant_to_sample, Median (IQR)	371 (185, 582)	1474 (822, 2142)	133 (73, 152)	376 (369, 378)	**0.002**
CNI[*], n (%)					1
FK506	19 (100)	6 (100)	6 (100)	7 (100)	

FK506 C_0 (ng/ml), Median (IQR)	6.5 (5.10, 7.80)	7.8 (7.58, 9.75)	5.5 (5.05, 6.30)	6.3 (5.55, 8.25)	0.168
White blood cell count (10^9/L), Median (IQR)	7.44 ± 2.14	7.63 ± 2.82	7.71 ± 2.25	7.05 ± 1.63	0.847
Lymphocyte count (10^9/L), Mean ± SEM	1.35 ± 0.63	1.07 ± 0.38	1.32 ± 0.66	1.61 ± 0.75	0.331
Neutrophil (10^9/L), Median (IQR)	5.41 ± 2.12	5.83 ± 2.63	5.70 ± 1.71	4.80 ± 2.14	0.657
Monocyte count (10^9/L), Mean ± SEM	0.57 ± 0.17	0.62 ± 0.17	0.58 ± 0.22	0.52 ± 0.14	0.608

CNIs, Calcineurin inhibitors; FK506, Tacrolimus; LD, Living donor; DD, Death donor. * Standard maintenance immunosuppression, including CNI, prednisolone and mycophenolic acid was applied to all patients.

Table S4. qPCR primer sequences.

Gene	Forward primer sequence 5'→3'	Reverse primer sequence 5'→3'
mouse-*β-Actin*	GGCTGTATTCCCCTCCATCG	CCAGTTGGTAACAATGCCATGT
mouse-*Tnfα*	TCTCATCAGTTCTATGGCCC	GGGAGTAGACAAGGTACAAC
mouse-*Granzyme B*	TCGACCCTACATGGCCTTAC	TGGGGAATGCATTTTACCAT
mouse-*Pd-l1*	TCTGATCGTCGATTGGCAGC	CGTTGTTCCAGGCTCCTCTC
mouse-*Fgl1*	CTTCGTCCTGGTCGCCATT	TCCCGCAAGCAGTTCTCAC
mouse-*Tim3*	TCAGGTCTTACCCTCAACTGTG	GGCATTCTTACCAACCTCAAACA
mouse-*Vista*	GACAGGTGGCCTCTCACC	TTTTCGATTCCTTGGGTGTT
mouse-*Ctla4*	GCTTCCTAGATTACCCCTTCTGC	CGGGCATGGTTCTGGATCA
mouse-*Tigit*	CTGATACAGGCTGCCTTCCT	TGGGTCACTTCAGCTGTGTC
mouse-*Il-12*	CAGCATGTGTCAATCACGCTAC	TGTGGTCTTCAGCAGGTTT
mouse-*Pd-1*	ACCCTGGTCATTCACTTGGG	CATTTGCTCCCTCTGACACTG
mouse-*LAG-3*	AGGTTCCAGGGTGGGGC	GTCCACTTGGCAGTGAGGAA
mouse-*Foxp3*	CACCTATGCCACCCTTATCCG	CATGCGAGTAAACCAATGGTAGA
human-*LAG-3*	TCACTGTTCTGGGTCTGGAG	GGTAAAGTCGCCATTGTCTC
human-*PD-1*	GCACGAGGGACAATAGGAGCC	AATGGTGGCATACTCCGTCTG
human-*ACTIN*	CCACACTGTGCCCATCTAC	AGGATCTTCATGAGGTAGTCAGTC
human-*FGL1*	AGTCTGCTTCTGCTACTTGG	TCCCTCCATCTTCACCCTTATT
human-*PD-L1*	GGTGAGGATGGTTCTACACAG	GAGAACTGCATGAGGTTGC
human-*TBET*	GCAGCACCGCTACTTCTACC	GTAGGCGTAGGCTCCAAGG
human-*GATA3*	AGAGCGTGGCCTGGAGAC	CTCCCACAGTCAAGGAAACC
human-*FOXP3*	GAGAAGCTGAGTGCCATGCA	TTGATCTTGAGGTCAGGGGCCAGG

human-*RORγt*	AAATCTGTGGGGACAAGTCG	CTGACGGGTGCAGGAGTAG
human-U6	CTCGCTTCGGCAGCACA	AACGCTTCACGAATTTGCGT
mQ-PRIMER-R	GTGCAGGGTCCGAGGT	
miR-let-7b-5p-F	GCGCTGAGGTAGTAGGTTGT	
miR-21-5p-F	GCGCTAGCTTATCAGACTGA	
miR-125b-5p-F	GCTCCCTGAGACCCTAAC	

Reference

[1] G. Zhu, L. Pei, F. Lin, H. Yin, X. Li, W. He, N. Liu, X. Gou, *J. Cell Physiol.* **2019**, *234* (12), 23736.

[2] R. Li, Q. Ruan, F. Yin, K. Zhao, *J. Pharmacol. Sci.* **2021**, *145* (1), 69.

[3] S. Banerjee, H. Cui, N. Xie, Z. Tan, S. Yang, M. Icyuz, V. J. Thannickal, E. Abraham, G. Liu, *J. Biol. Chem.* **2013**, *288* (49), 35428.

[4] T. Bahmer, S. Krauss-Etschmann, D. Buschmann, J. Behrends, H. Watz, A. M. Kirsten, F. Pedersen, B. Waschki, O. Fuchs, M. W. Pfaffl, E. von Mutius, K. F. Rabe, G. Hansen, M. V. Kopp, I. R. Konig, S. Bartel, *Allergy* **2021**, *76* (1), 366.

[5] X. Wang, H. X. Wang, Y. L. Li, C. C. Zhang, C. Y. Zhou, L. Wang, Y. L. Xia, J. Du, H. H. Li, *Hypertension* **2015**, *66* (4), 776.

[6] M. Reis, E. Mavin, L. Nicholson, K. Green, A. M. Dickinson, X. N. Wang, *Front. Immunol.* **2018**, *9*, 2538.

[7] J. Liu, L. Fan, H. Yu, J. Zhang, Y. He, D. Feng, F. Wang, X. Li, Q. Liu, Y. Li, Z. Guo, B. Gao, W. Wei, H. Wang, G. Sun, *Hepatology* **2019**, *70* (1), 241.

[8] S. E. Nematian, R. Mamillapalli, T. S. Kadakia, M. Majidi Zolbin, S. Moustafa, H. S. Taylor, *J. Clin. Endocrinol. Metab.* **2018**, *103* (1), 64.

[9] J. Y. Cao, B. Wang, T. T. Tang, Y. Wen, Z. L. Li, S. T. Feng, M. Wu, D. Liu, D. Yin, K. L. Ma, R. N. Tang, Q. L. Wu, H. Y. Lan, L. L. Lv, B. C. Liu, *Theranostics* **2021**, *11* (11), 5248.

[10] Y. Y. Yang, X. X. Zuo, H. L. Zhu, S. J. Liu, *Beijing Da Xue Xue Bao Yi Xue Ban* **2019**, *51* (2), 374.

[11] G. Guo, H. Wang, X. Shi, L. Ye, K. Wu, K. Lin, S. Ye, B. Li, H. Zhang, Q. Lin, S. Ye, X. Xue, C. Chen, *J. Transl. Med.* **2018**, *16* (1), 370.

[12] a) H. Abken, *Transplantation* **2021**, *105* (7), 1394; b) S. Sad, T. R. Mosmann, *J. Immunol.* **1994**, *153* (8), 3514.

[13] M. Sawada, T. Kawayama, H. Imaoka, Y. Sakazaki, H. Oda, S. Takenaka, Y. Kaku, K.

Azuma, M. Tajiri, N. Edakuni, M. Okamoto, S. Kato, T. Hoshino, *PLoS One* **2013**, *8* (1), e54623.

[14] T. J. Scriba, B. Kalsdorf, D. A. Abrahams, F. Isaacs, J. Hofmeister, G. Black, H. Y. Hassan, R. J. Wilkinson, G. Walzl, S. J. Gelderbloem, H. Mahomed, G. D. Hussey, W. A. Hanekom, *J. Immunol.* **2008**, *180* (3), 1962.

[15] a) J. Niu, W. Yue, Y. Song, Y. Zhang, X. Qi, Z. Wang, B. Liu, H. Shen, X. Hu, *Clin. Exp. Immunol.* **2014**, *176* (3), 473; b) C. I. Kingsley, M. Karim, A. R. Bushell, K. J. Wood, *J. Immunol.* **2002**, *168* (3), 1080.

[16] M. Goldman, A. Le Moine, M. Braun, V. Flamand, D. Abramowicz, *Trends in immunology* **2001**, *22* (5), 247.

[17] M. P. Crawford, S. Sinha, P. S. Renavikar, N. Borcherding, N. J. Karandikar, *Proc. Natl. Acad. Sci. U. S. A.* **2020**, *117* (32), 19408.

[18] a) C. Jandl, S. M. Liu, P. F. Canete, J. Warren, W. E. Hughes, A. Vogelzang, K. Webster, M. E. Craig, G. Uzel, A. Dent, *Nat. Commun.* **2017**, *8* (1), 1; b) A. Suto, D. Kashiwakuma, S. Kagami, K. Hirose, N. Watanabe, K. Yokote, Y. Saito, T. Nakayama, M. J. Grusby, I. Iwamoto, H. Nakajima, *J. Exp. Med.* **2008**, *205* (6), 1369.

[19] G. Trinchieri, S. Pflanz, R. A. Kastelein, *Immunity* **2003**, *19* (5), 641.

[20] A. Kimura, T. Kishimoto, *Eur. J. Immunol.* **2010**, *40* (7), 1830.

[21] D. B. Hoelzinger, S. E. Smith, N. Mirza, A. L. Dominguez, S. Z. Manrique, J. Lustgarten, *J. Immunol.* **2010**, *184* (12), 6833.

[22] T. Maruyama, J. Li, J. P. Vaque, J. E. Konkel, W. Wang, B. Zhang, P. Zhang, B. F. Zamarron, D. Yu, Y. Wu, Y. Zhuang, J. S. Gutkind, W. Chen, *Nat. Immunol.* **2011**, *12* (1), 86.

[23] C. Liu, H. C. Wang, S. Yu, R. Jin, H. Tang, Y. F. Liu, Q. Ge, X. H. Sun, Y. Zhang, *J. Immunol.* **2014**, *193* (2), 663.

[24] H. Nishikii, B. S. Kim, Y. Yokoyama, Y. Chen, J. Baker, A. Pierini, M. Alvarez, M.

Mavers, K. Maas-Bauer, Y. Pan, S. Chiba, R. S. Negrin, *Blood* **2016**, *128* (24), 2846.

[25] M. Kuczma, P. Kraj, *Vitam. Horm.* **2015**, *99*, 171.

[26] B. A. Jones, M. Beamer, S. Ahmed, *Mol. Interv.* **2010**, *10* (5), 263.

[27] A. Curti, A. Tafuri, M. R. Ricciardi, P. Tazzari, M. T. Petrucci, M. Fogli, M. Ratta, R. Lapalombella, E. Ferri, S. Tura, M. Baccarani, R. M. Lemoli, *Haematologica* **2002**, *87* (4), 373.

[28] S. M. Metcalfe, *Genes Immun.* **2011**, *12* (3), 157.

[29] B. P. Lee, W. Chen, H. Shi, S. D. Der, R. Forster, L. Zhang, *J. Immunol.* **2006**, *176* (9), 5276.

[30] S. A. Joosten, K. E. van Meijgaarden, N. D. Savage, T. de Boer, F. Triebel, A. van der Wal, E. de Heer, M. R. Klein, A. Geluk, T. H. Ottenhoff, *Proc. Natl. Acad. Sci. U. S. A.* **2007**, *104* (19), 8029.

[31] Z. Y. Zhou, S. L. Chen, N. Shen, Y. Lu, *Autoimmun Rev.* **2012**, *11* (10), 699.

[32] F. Shi, Y. Xiong, Y. Zhang, C. Qiu, M. Li, A. Shan, Y. Yang, B. Li, *Inflammation* **2018**, *41* (3), 1021.

[33] A. de Luca, S. Bozza, T. Zelante, S. Zagarella, C. D'Angelo, K. Perruccio, C. Vacca, A. Carvalho, C. Cunha, F. Aversa, L. Romani, *Cell Mol. Immunol.* **2010**, *7* (6), 459.

[34] S. Abdollahi-Roodsaz, L. A. Joosten, M. I. Koenders, I. Devesa, M. F. Roelofs, T. R. Radstake, M. Heuvelmans-Jacobs, S. Akira, M. J. Nicklin, F. Ribeiro-Dias, W. B. van den Berg, *J. Clin. Invest.* **2008**, *118* (1), 205.

[35] J. C. Waite, D. Skokos, *Int. J. Inflam.* **2012**, *2012*, 819467.

[36] N. Takemoto, A. M. Intlekofer, J. T. Northrup, E. J. Wherry, S. L. Reiner, *J. Immunol.* **2006**, *177* (11), 7515.

[37] C. S. Hinrichs, R. Spolski, C. M. Paulos, L. Gattinoni, K. W. Kerstann, D. C. Palmer, C. A. Klebanoff, S. A. Rosenberg, W. J. Leonard, N. P. Restifo, *Blood* **2008**, *111* (11), 5326.

[38] X. Sun, H. Yamada, K. Shibata, H. Muta, K. Tani, E. R. Podack, Y. Yoshikai, *J. Immunol.*

2010, *185* (4), 2222.

[39] L. Xia, L. Jiang, Y. Chen, G. Zhang, L. Chen, *Cytokine* **2021**, 155658.

[40] P. G. Miller, M. B. Bonn, S. C. McKarns, *J. Immunol.* **2015**, *195* (6), 2633.

[41] M. Kitajima, H. C. Lee, T. Nakayama, S. F. Ziegler, *Eur. J. Immunol.* **2011**, *41* (7), 1862.
